# Revealed Preference Methods for Studying Bicycle Route Choice—A Systematic Review

**DOI:** 10.3390/ijerph15030470

**Published:** 2018-03-07

**Authors:** Ray Pritchard

**Affiliations:** Department of Architecture and Planning, Faculty of Architecture and Design, NTNU—Norwegian University of Science and Technology, 7491 Trondheim, Norway; raymond.pritchard@ntnu.no

**Keywords:** bicycle, bicycle route choice, revealed preference, naturalistic, built environment, physical activity, route choice model

## Abstract

One fundamental aspect of promoting utilitarian bicycle use involves making modifications to the built environment to improve the safety, efficiency and enjoyability of cycling. Revealed preference data on bicycle route choice can assist greatly in understanding the actual behaviour of a highly heterogeneous group of users, which in turn assists the prioritisation of infrastructure or other built environment initiatives. This systematic review seeks to compare the relative strengths and weaknesses of the empirical approaches for evaluating whole journey route choices of bicyclists. Two electronic databases were systematically searched for a selection of keywords pertaining to bicycle and route choice. In total seven families of methods are identified: GPS devices, smartphone applications, crowdsourcing, participant-recalled routes, accompanied journeys, egocentric cameras and virtual reality. The study illustrates a trade-off in the quality of data obtainable and the average number of participants. Future additional methods could include dockless bikeshare, multiple camera solutions using computer vision and immersive bicycle simulator environments.

## 1. Introduction

The promotion of cycling is increasingly seen as an effective and efficient tool for reducing the negative environmental impacts of transport whilst improving quality of life [1]. By enabling a shift from motorised transportation to cycling, cities can reduce both their greenhouse gas contribution and improve regional air quality through reduced motorised transportation [2]. Increasing cycling rates in this manner has been demonstrated to have substantial health benefits, despite the increased exposure to air pollution and traffic [3].

Traditionally transport planners have made use of such techniques as manual traffic volume counts at set points in a traffic network to create traffic demand models (for all modes of transport). Today, count data remains valuable in many respects and a wide variety of automated sensor technologies are available to provide continuous information on traffic flows. This type of data collection is not within the scope of this article, since it does not reveal details about bicyclist trip lengths, infrastructure preferences or network behaviour. However reviews and evaluations of the available technologies for the volume counting of bicycles can be found from the US National Cooperative Highway Research Program (NCHRP) [4,5] and select other sources [6,7,8].

This research systematically reviews the scientific literature for data collection techniques that allow researchers and planners to understand the route choice behaviour of bicycle users. It builds and expands upon earlier research concerning information technology dependent means for determining the location of physical activity—by considering also more traditional methods that have been used to determine bicycle route choice. Krenn et al. [9] conducted a review of GPS studies in the scientific and grey literature that examine physical activity. Loveday et al. [10] similarly explore the use of GPS in a comprehensive review of wearable or portable technologies that measure location. Buehler & Dill [11] conduct a review of the literature concerning the evaluation of bicycle networks and other bicycle infrastructure, meaning that a number of less technology dependent methods were uncovered. Lastly, a review by Romanillos et al. [12] considered all big data technologies associated with cycling—covering GPS, crowdsourcing and smartphone related methods together with live point data and origin-destination data. In contrast to other review papers, this paper includes bicycling for all trip purposes, focuses on all methods that can be applied to the empirical determination of whole journey route choice and covers digital publications from all years up until late 2017. Whole journey route choice refers specifically to the route choice along an entire origin-destination journey, turn-by-turn.

Route choice data based on actual cycling behaviour is well suited to context-specific applications such as the evaluation of new infrastructure, safety assessments or pollutant exposure. It should however be noted that tracing the whole journey of cyclists is not an entirely new endeavour. Individual travel surveys often request participants to recall recently traversed routes and the transport mode used. In recent years, GPS technology has become very affordable and increasingly omnipresent, allowing its use in large studies on travel behaviour. The goal of this paper is to compare the traditional and newer techniques that have been applied to the study of bicycle route selection.

## 2. Methods

This paper systematically reviews the revealed preference methods that have been applied to the study of whole-journey bicycle route choice. Empirical data on bicycle route choice can also be collected through aggregate volume measures (like heat maps from the aggregated tracking of multiple users) and through naturalistic studies of point locations such as observations at intersections. Additionally stated preference techniques are often used for hypothetical route choice, where respondents are presented with a series of choices to compare against a trade-off such as time or cost. However, since such techniques do not review the full journey of the individual decision maker, the bicycle user, they are not covered in this review. The process through which the literature has been identified is described in detail below.

**Figure 1:**
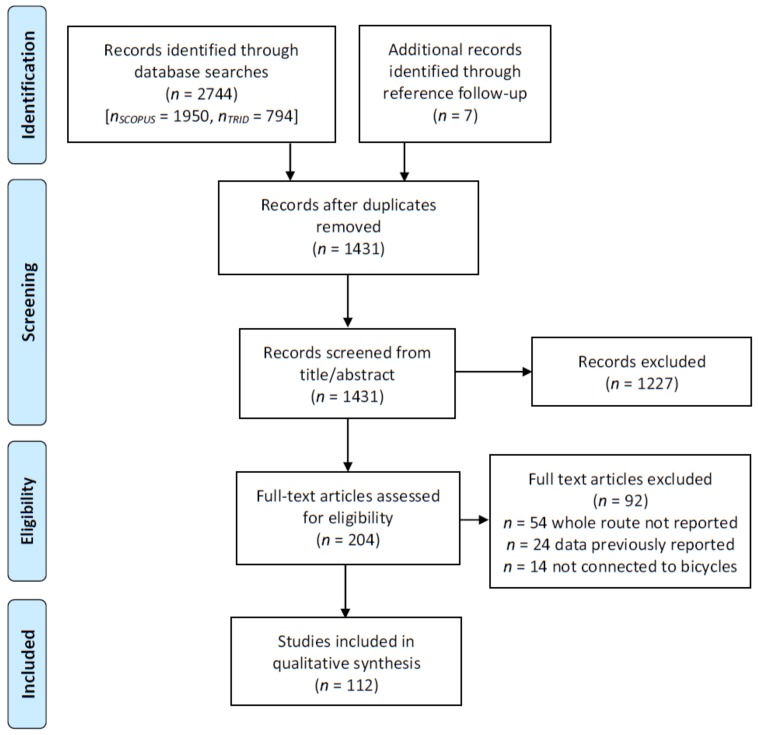
Pathway diagram of included and excluded articles in review.

To identify sources, searches were made in December 2017 in the Scopus (Elsevier) and Transport Research International Documentation (TRID) databases. The TRID database is a combination of the Transportation Research Board’s Transportation Research Information Services database and the Joint Transport Research Centre’s International Transport Research Documentation Database maintained by the Organisation for Economic Co-operation and Development. The TRID database importantly contains transport-related theses and grey literature such as reports that are not published in Scopus or many other journal databases. Only English language records were reviewed with no publication date restrictions, using the query “route choice” or “naturalistic” or “revealed preference” in combination with any of the strings bicycl*, bik* or cycl* (where the asterisk indicates all iterations hereafter). Records were required to be available in digital format to be included in this review. The search strategy is summarised in Figure 1 below [13].

In total, 112 empirical studies were uncovered by this search strategy. The principal selection criterion for empirical studies was that the methods had been applied to the study of bicycle route choice, and that the whole journey is captured by the method. The majority of these empirical studies were published in journals or as book chapters (65), followed by conference proceedings (30), reports (14) and theses (3).

The following section presents the results, or introduction to the literature. This is followed by the discussion in which the timeline of publications, geographical distribution of research and the numbers of participants for different method families are displayed.

## 3. Results

**Figure 2:**
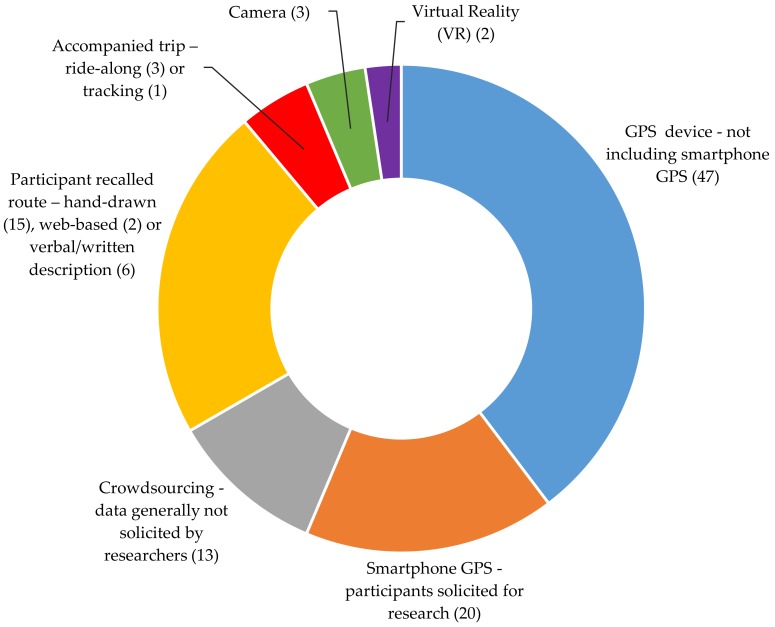
Seven method families for categorisation of the literature (numbers of articles in parentheses).

The results section of this paper is structured as follows. Firstly, the primary method families for classification purposes is explained. This is graphically illustrated in Figure 2, which displays the numbers of articles categorised in each family. Families are defined according to the primary method used to ascertain location/route choice, although in many studies, multiple methods are used that could provide this information. Detailed findings are subsequently discussed, with the further breakdown of the seven method families into 34 sub-groups. This second level of classification according to research design rather than method is used to ensure that no one sub-group contains more than ten studies, simplifying the summary of findings. A tabularised summary of the literature using this method family and sub-group structure is introduced in Appendix A, Table A1. Because many of the studies utilise multiple methods, the Table A1 includes a column qualitatively indicating the frequency of method combination for each sub-group. The other methods are not restricted to route choice, but may be supplementary data sources such as accelerometer measurements, heart rate monitors or cameras. More checks in the ‘integration with other methods’ column indicates more frequent combination with other methods.

Method families must have a substantially different methodological set-up to other families. Hence, even though the first three families make use of GPS technology, they are split into separate families due to differences in research design (researcher acquisition of GPS device, participant GPS ownership through smartphone, and collection of crowdsourced GPS data). A summary of the literature in each family is made using the same structure (sub-groups) as in Table A1. In many cases, empirical studies make use of two or more methods for ascertaining route choice, however only the principal method (or the method that is considered most important for determining route choice) is used for classification in Table A1.

### 3.1. GPS Devices

A total of 47 articles were found that discuss unique data collection efforts using Global Positioning System (GPS) devices (excluding smartphone GPS and crowdsourcing studies but inclusive of GPS integrated into other devices such as helmet cameras or sports watches). A study of the accuracy of Smartphone GPS relative to enhanced GPS units demonstrated that whilst GPS devices were significantly more accurate than smartphone GPS, no statistically significant difference was found between smartphone manufacturers [14]. Additionally other data sources are required to be able to determine the street position of cyclists (on bicycle lane, footpath or traffic lane).

The frequency of geo-located point provision and thus geospatial accuracy of GPS devices was reported in 21 of the 47 studies. Frequency values ranged from 0.2 Hz [15] to 10 Hz [16,17,18], with a median of 1 Hz (or one position located per second). Such frequencies were not experienced to be problematic for recording route choices, although other issues including lack of signal, inaccurate positioning or loss of battery power were significant causes of data loss [9]. The review article from Krenn et al. [9] also reports data loss issues concerning charging of GPS units for eight of 24 included studies.

Missing data was also a problem for a longitudinal study of children’s school journeys in northern England conducted in 2007 where an estimated 39% of journeys were not recorded due to problems obtaining an initial position fix from satellites [19]. One Portland-based study used a Personal Digital Assistant (PDA) with GPS functionality, meaning that it was simple to set up to ask travel survey questions prior to beginning or finishing a trip segment [20]. The user interface was a contributing element to getting participants to check battery charge, resulting in the relatively low data loss of 8% [20].

Most of the 34 studies that utilise an instrumented bicycle setup (two or more devices attached to either a research-team or participant-owned bicycle) utilised GPS devices rather than smartphones. As might be expected, the use of portable GPS loggers improves satellite fix and accuracy in comparison to smartphone-embedded GPS units [14]. It should be noted however that weatherproofing of GPS devices and other instrumentation (or failure to!) for use in longer terms studies can also create signal issues. Thus, special consideration should be given to this during testing phases of future research projects.

Three of the earliest studies, published between 2007 and 2010 found that data collection was very time intensive for the research teams due to the need to extract data every few days from the units. [19,20,21]. This became less of an issue in subsequent research that made use of wireless mobile data transmission [22,23], shorter data collection periods such as for test tracks or predetermined routes [17,24,25,26,27,28] or simply had larger memory cards [29].

Since many of the studies published after 2010 use largely similar GPS devices, the remaining studies are discussed according to five sub-groups, as outlined in Table A1. Each sub-group has sufficiently different methodological design to justify a distinction, with the intent to have no more than 10 articles per sub-group. It should be noted that the distinction between what constitutes an “instrumented” set-up as opposed to a standard GPS study was small, however for this paper “instrumented” refers to studies in which two or more separate devices are carried or mounted to a bicycle/vehicle.

#### 3.1.1. Instrumented Research Bicycles/Pedelecs

Instrumented research bicycles were generally loaned out to participants to obtain data over relatively short time periods, and are sometimes also referred to as bicycle Data Acquisition Systems [30,31] or Instrumented Probe Bicycle [25]. Instrumented research bicycles were used in studies of both conventionally powered [17,25,26,30,32,33,34,35,36] and pedal-assisted electric bicycles (pedelec) [16,22,24,28,37]. The bicycles are loaned to participants in a configured state (with GPS as the primary means of determining route choice), and the use of a single bicycle type can have benefits when observing such phenomena as steering, overtaking distances or acceleration/vibration [25,26,36,37]. This is because minor differences in suspension and steering between different bicycle models are removed as a confounding factor. Instrumented research bicycles and pedelecs are used most often in the context of specific research designs, usually focussing on a specific area or even fixed route. Thus participants are generally only required to cycle for a limited time—typically one to two trips. An exception was a pedelec study in which participants were loaned an instrumented bicycle for a period of two weeks [16].

#### 3.1.2. Instrumented Participant Bicycles/Pedelecs

Other instrumented bicycle studies made use of similar experimental set-ups mounted to participants’ conventional bikes [27,31,38,39,40,41]. Some of these studies required participants to use their bicycles in an instrumented form for a week or more [39,41], whilst the others were similar to the instrumented research bicycles in their experimental design, requiring users to make only one to two trips. A smaller number of studies instrumented participants’ pedelec bicycles, for between 4 and 30 weeks. The four week study investigated the behaviour of both conventional bicyclists (*n* = 31), pedelec users (*n* = 49) and higher-powered s-pedelec users (*n* = 10) in Germany [42]. The longer term 30 weeks study occurred amongst 61 pedelec users in Ghent, Belgium but without any additional user involvement such as through the completion of a travel diary [15]. Long-term measurements were enabled by having automatic activation when the pedelec is in use, and whilst neither study specified charging routines for the instrumentation, it could be expected that the use of the pedelec battery would significantly reduce the effort required of participants.

#### 3.1.3. Instrumented Quasi-Bikeshare

A third type of instrumented bicycle study incorporating GPS devices is for quasi bikeshare schemes. Unlike public bikeshare in which any paying member of the public can use a bicycle, quasi bikeshare is available only for a subset of the population. In this review, two studies were uncovered that discuss pilot bikeshare schemes for exclusive use within university environments. One was implemented at the University of Tennessee, USA in which seven pedelecs and six conventional bicycles in the same bikeshare system were configured with GPS devices for the use of around 100 mostly undergraduate student users [43]. The other study discusses a university bikeshare system at UMONS in Belgium but only a detailed description of the proposed sensor configuration to be implemented was included [23]. It should be noted that GPS equipped bikeshare bicycles used in bicycle route choice research are categorised according to research design rather than bicycle type, resulting in two more bikeshare papers being discussed in results Section 3 [44,45].

#### 3.1.4. Instrumented Participant (Two or More Devices)

Four studies make use of a collection of instruments, but as wearable devices rather than bicycle-mounted systems. All of these studies required participants to carry instrumentation with them for a minimum of one week. A longitudinal study of children’s school route and mode choice in northern England made use of a smartphone with external GPS receiver to allow annotation of journeys as they are made [19]. A pollution focused study in Colorado USA made 45 participants travelling with all modes to wear a modified backpack containing GPS together with air intake tubes connected to air quality instrumentation [46]. Lastly two before and after infrastructure evaluation studies in Portland [47] and Salt Lake City, USA [48] required 341 and 939 participants respectively to wear both a GPS device and accelerometer.

#### 3.1.5. Participant-Borne/Wearable GPS Devices

Other wearable GPS devices included helmet cameras with built in GPS functionality [49,50,51], whilst all bar one of the remaining studies used GPS devices that were either wearable [20,52,53,54,55,56], participant-borne [18,29,57,58,59,60,61] or mounted to participants’ bicycles/vehicle [62].

GPS device data have been collected much earlier in the context of travel surveys, but these studies tend not to contain the search terms used by this paper [63,64,65].

### 3.2. Smartphone Applications

There are 20 papers that discuss the use of smartphone applications to capture travel behaviour, of which 16 specifically collect data related to cyclists, two are for all transport modes, and two are for vulnerable road users. Broadly speaking, smartphone applications for route choice studies can be split into two categories of passive or active user registration. Passive smartphone studies require mode identification or be involved in instrumented research bicycle set-ups where mode is no longer a variable. Active studies meanwhile tend to focus solely on cycling, and require the user to manually start and stop GPS logging via the application interface. This section will start by discussing generic issues related to smartphone applications followed by a focus on passive and active smartphone apps.

Frequency of data provision was reported by five of the smartphone-based studies, all of which recorded data at 1 Hz (or one position located per second). It should be noted that four of these studies were in association with active applications [66,67,68,69], whilst the remaining study used a smartphone in a long term instrumented pedelec study [70]. The instrumented pedelec research group sought to actively minimised battery drain, despite the connection of the instrumentation to the large pedelec battery. This was achieved by recording the accelerometer and GPS readings for the first four seconds of every minute, and using this information to determine if the bicycle was in motion. When no motion was recorded, the smartphone would return to a low energy sleep mode. Active smartphone applications however require the user to start and stop GPS recording. The total length of active GPS time is strongly correlated with battery consumption, however because it is mostly inactive, battery use was rarely reported to be a concern. One exception was for an active smartphone study that also included a bicycle courier sub-group, who could be expected to flatten a smartphone battery when recording for a full day of cycling [71]. The solution was to provide this group dedicated GPS devices with larger battery capacity. Thus battery concerns are mostly restricted to the passive smartphone application subset, however only the aforementioned instrumented pedelec study mentioned battery concerns.

Similar to GPS devices, smartphones can experience connectivity issues in obtaining a satellite fix. Such problems can be worse because the devices are not specifically designed to provide optimum location information, but also because the GPS sensor in the mobile is frequently shielded by clothes or items in a bag. The connectivity issues in smartphones can be alleviated through combination with other integrated sensors such as cellular network location [72]. Wearable and bicycle-mounted options minimise shielding, increasing chances of a fast connection to satellites.

#### 3.2.1. Passive Smartphone Application

The oldest paper describes the development and pilot testing of a smartphone application called TRAC-IT, which explores the potential to replace traditional travel surveys [72]. It additionally investigates techniques for mode detection and the practicality of a critical points algorithm, intended to reduce data transfer requirements to that which is necessary for reconstructing a route. A similar study specifically for Blackberry phones sought to develop a mode classifier integrated with trip segmentation using 658 verified trips [73]. This was the only smartphone application study not to use either Google Android or Apple’s iPhone Operating System (iOS).

A separate passive study focussed on the development of an app called LogYard, for automatic crash notification for vulnerable road users and in particular all-terrain vehicle users [66]. The authors tested the concept in a pilot study on cyclists, which, after the collection of simulated crash and bicycle movement data, demonstrated an algorithm that could detect accidents.

#### 3.2.2. Passive Smartphone Application for Instrumented Research Bicycle/Pedelec

An instrumented research bicycle set up similar to those mentioned in results section 1 was used in four studies, three of which were implemented on pedelecs. All of the pedelec studies utilised wireless data transfer, whilst a fourth instrumented bicycle study used a conventional bicycle with four helmet-mounted video cameras, requiring manual data download. The conventional instrumented bicycle was used in a fixed route experiment by researchers in Portland [68]. In addition to the helmet cameras and 1 Hz smartphone GPS location, the experiment also included a galvanic skin response stress sensor and a power meter. In total six subjects rode the instrumented bicycle at different times of day in order to ascertain the impact of variable traffic levels.

A similar fixed route setup was made in Austria, with the instrumentation of two different types of electric bicycles in combination with a test of electrically and conventionally powered mopeds [74]. The e-bike experiment collected data from altogether 145 participants, with a fish-eye camera complementing the smartphone-integrated GPS. A custom-designed app allowed remote operator control of the instrumented bicycle via a Wireless Local Area Network base station at the experiment site.

A larger study in Brighton, UK equipped a fleet of 35 e-bikes with a smartphone and power assistance sensors in a so-called Smart E-bike Monitoring System (SEMS) [67]. SEMS was powered by the e-bike battery and saved energy by running in a low power sleep state for the majority of the time. Every 25 s the phone woke and queried the accelerometer for 1.5 s. The system was kept active if movement was detected, otherwise the phone returned to the sleep state. Ninety-three participants took ownership of the research bicycles for up to eight weeks, and were provided with real-time feedback about their cycling activity via an online portal.

A year-long field trial of 31 smartphone-instrumented electric bicycles was performed at the University of Waterloo in Canada [70]. Unlike most instrumented research designs, the pedelecs were given to users in return for their participation, and were not rotated amongst a larger pool of participants. External sensors were limited to battery usage and performance, whilst discharge of the battery was minimised by keeping the phone in a sleep mode most of the time, only querying location and battery indicators for the first four seconds of every minute.

#### 3.2.3. Existing Active Smartphone Applications 

Active user smartphone apps are commonly used in the literature, where users are required to manually start and stop GPS recording. One benefit of active user apps is that the frequency of GPS recordings can be relatively high without battery depletion concerns (where passive apps frequently need to optimise energy use through reduced sensitivity). Several of the apps are specifically designed for research and planning purposes, such as CycleTracks, originally developed by the San Francisco County Transportation Authority and used in a five month study of 1083 users in the same city [75]. GPS data is collected and stored locally on the device, with uploads data upon completion of a trip. The same application was also used in studies of three other US cities: Austin, Texas with 317 bicyclists [76], Seattle with 197 bicyclists over a 3.5 years period [77] and Columbus, Ohio with 76 cyclists [78]. The CycleTracks application was developed with specific consideration to minimise battery drain whilst in use, turning off when the phone battery level reaches 10% [76].

The success of the CycleTracks application in multiple studies lead to its replication in a number of other regions, who built their own version of the application based on the original code. Three studies of this nature were found including: ORCycle in Oregon with 381 users [79], CycleAtlanta with 1529 users [69] and CycleLane in Eugene, Oregon with 103 users [80].

One smartphone study made use of the recreational tracking application Map My Tracks to collect the GPS traces of cyclists and bike couriers in Madrid [71]. The study required participants to upload their tracks collected in the tracking application or with GPS devices to either the project website or application, making the required level of involvement from users higher than most other studies in this category. Note that studies that directly obtain data from sports or recreational applications without researcher input, are considered by this study to be crowdsourcing, and are discussed in the next section.

#### 3.2.4. Other Customised Active Smartphone Applications

A similar tailor-made app was commercially developed for use by the City of Toronto, confusingly called CycleTrack, which achieved a high level of participation: 4556 users and 33,220 journeys recorded over nine months [81]. Interestingly over half of participants reported that they had not cycled since they were children, a contrast from most other smartphone-based studies, whose participants were mostly experienced riders.

Other customised apps includes the Mon RésoVélo smartphone application created by McGill researchers, who obtained 10,000 trips from nearly 1000 users in Montreal, Canada, during four months of 2013 [82]. BeCity is a customised application built in same manner as the sports monitoring application Endomondo, but provides users with routing feedback due to the lack of other commercial actors performing this for cycling [83]. Other customised smartphone apps for research or planning purposes were implemented in Gothenburg, Sweden, with 15 bicyclists instructed to ride on selected routes [84] and in a route choice model study using data from 774 cyclists for Transport for London [85]. These studies did not provide details concerning the application development or frequency of geo-located positions.

### 3.3. Crowdsourcing

Thirteen papers make use of crowdsourcing as the principal method for obtaining location, amongst which ten utilised smartphone GPS applications and the one remaining study used a crowdsourcing platform for GPS devices. The ten crowdsourcing smartphone studies are split into sports applications (Strava, Sports Tracker and Endomondo), research/planning-oriented smartphone applications (Fiets Telweek, BikePRINT, RiderLog), a citizen science platform (Amazon Mechanical Turk) and individually donated GPS logs.

#### 3.3.1. Recreational/Sports Applications

Three studies included in this paper make use of the bicycle training oriented smartphone application Strava and the associated paid service StravaMetro, however none showed the full routes of individuals. Instead the studies made use of origin-destination data, link/street counts or node counts [86,87,88]. This is because of StravaMetro’s policy of data aggregation whereby it is not possible to see how any individual route looks, most likely due to the privacy interests of users. Despite not meeting the inclusion criteria for this paper, Strava was included nonetheless because of the size of the dataset, the potential for individual route data to become available (it is deliberately reduced in quality) and because individual users can still opt to donate their routes to researchers. One such data donation initiative is the Bike Data Project, started after the 2015 release of Frederick Gertten’s Bikes Vs Cars documentary, which easily links to the user accounts of three sports applications Moves, Runkeeper and Strava (http://bikedataproject.com). Whilst Strava does not presently provide the individual route traces as a crowdsource, it has been used as a supplementary method by researchers for ride-along and ethnographic studies. More information on ride-along as a method is found in results Section 3.5.1. Lastly, it should be noted that Strava also allows for the input of GPS data from bicycle computers and GPS devices other than smartphones.

A similar mobile application called Sports Tracker was used in a separate study focussed on providing automatic popularity-based routing in Helsinki [89]. In this study, the full route trace of individuals was used, with a public dataset of nearly 30,000 routes from 1994 users. An issue witnessed by the researchers was the skewed distribution of routes, where 5% of the users had recorded 50% of the tracks. High variation in participation is however an issue across the crowdsourcing studies, and effects also the studies using GPS devices [58].

Endomondo, the final sports application to be included here, was used in the context of the European Cycling Challenge (ECC) in 2013 [90]. The ECC is an annual initiative in the month of May that seeks to gamify cycling across participating European cities (http://www.cyclingchallenge.eu). The Bologna dataset obtained by the researchers contained no information about the numbers of users, but approximately 5900 routes were available in the raw data. Subsequent years of the ECC have used different mobile tracking applications, but in general, the cities participate for a nominal fee on the basis that they subsequently own the data collected by their residents. The ECC website displays heat maps of different cities and a leader board of top cities (per capita and in total) to gamify the experience and motivate use, whilst at the end of May the top cities are presented prizes.

The study that did not use smartphones instead gathered crowdsourced data from GPS devices (such as sports watches and cycling computers) through the recreation-oriented web platform RouteYou, a data sharing platform to enable users to find appropriate routes for recreational travel [91]. In this study, 190,610 bicycle-related records were collected over two years from 6300 unique users living in East Flanders, Belgium.

#### 3.3.2. Customised Applications for Nationwide Data Collection

A practice-oriented smartphone application called RiderLog was used by researchers in Sydney to validate an agent-based model and census data for the same region [92]. RiderLog is similar to the ECC in terms of goals, but rather than utilising a commercial application was specifically developed for the Australian Bicycle Network. The application is intended to stimulate cycling as an active transportation mode and provides users with a platform to monitor their progress.

Fiets Telweek, or Bicycle Counting Week, is an initiative from The Netherlands (similarly performed in Flanders) to crowdsource cycling data to better understand the behaviour of Dutch cyclists. The event has occurred for a single week each September since 2015, and it was this first year of data that was used in one study of Amsterdam cyclists [93]. The Amsterdam dataset available to the researchers included 12,413 trips from around 5000 users, and approximately one quarter of these trips were subsequently used in their creation of a discrete choice model for cyclist behaviour.

Prior to the start of Fiets Telweek in 2015, BikePRINT was developed to make use of smartphone GPS data through a custom-designed app, whilst displaying data in an interactive map that made it more user friendly [94]. The graphical interfaces demonstrated in the article do not reveal individual routes, but neither is it explicitly stated that the data is aggregated like Strava. Unfortunately, little detail is given about the process of data collection, however BikePRINT’s commercial successor The Urban Future (a spinoff from the NHTV Breda University of Applied Sciences), is at the time of writing hosting the 2015 to 2017 data for Fiets Telweek (http://app.cycleprint.eu).

#### 3.3.3. Volunteered (Post-Collection) Data

A small study collected volunteered GPS log files from both smartphones and GPS devices via Korean bicyclist groups [95]. Data collection efforts here demonstrated a greater representation bias than other crowdsourcing studies, as it focused only on enthusiast cyclists, all 54 of whom were male and aged between 19 and 42. Should the aforementioned Bike Data Project or similar platforms supporting volunteered data donation grow to represent many users, these representation problems could disappear.

#### 3.3.4. Instrumented Public Bikeshare

Two studies made use of instrumented bicycles in regular bikesharing schemes, whereby the first study retrofitted 130 Capital Bikeshare bicycles in Washington DC with GPS units [44]. The devices were retrieved after four weeks deployment, during which time 36 GPS units were lost, together with their data. The recovered units recorded data for two weeks on average prior to the battery running out, and recorded in total 3596 trips. Loss of trip data was avoided in the second bikeshare study due to the use of real-time location from the GPS-enabled Grid Bikeshare in Phoenix, USA [45]. The frequency of GPS readings was, however, relatively low, varying from one per minute to 25 per minute; a frequency sufficient for bikeshare fleet operators but not always sufficient for bicycle route analysis.

Although no studies in this review made use of dockless bicycles, their increasing presence in cities around the world warrants their brief mention. Public dockless bikeshare systems use GPS-enabled bicycles in distributed fleets without specific docking stations. GPS is a necessity for the system to work since users can locate bicycles in real time via a smartphone application. Like the Grid Bikeshare study, data from dockless bicycles could potentially be obtained via fleet operators, representing a very significant future source of empirical route choice data.

#### 3.3.5. Citizen Science Crowdsourcing Platform

Finally, a pilot demonstration of Amazon Mechanical Turk (AMT), a citizen science crowdsourcing platform, was used to gather participants for a smartphone GPS study [96]. Ten participants are reported on in this paper, however the research project aimed to collect data for 200 participants in total. AMT is a platform matching a large pool of workers and employers (called ‘requesters’) to perform relatively simple tasks such as data categorisation or image labelling. The researchers collected data in this manner for a payment level of $5, requiring participants to install a smartphone app, use the app for three days, upload the trip data, answer a survey and finally recruit somebody outside of the AMT network to do the same [96]. Although a large number of potential workers is available, they are geographically dispersed, meaning that findings collected from participants may not be representative for a particular city or region. Qualitative research may however find the disperse worker pool to be of an advantage for comparative studies of cycling environments.

### 3.4. Participant Recalled Route—Hand-Drawn, Web-Based or Verbal/Written Description 

#### 3.4.1. Hand-Drawn (Paper-Based) Route Collection

The collection of hand-drawn route data requires very few resources, making it highly versatile for implementation in various studies, most commonly in combination with interviews or paper-based surveys, distributed in various manners. Many of the 15 studies in this category use a largely similar research design, thus in the interests of brevity, only the major methods are discussed in this category, whilst the remainder have been summarised in Table A2.

The first study using hand-drawn routes was conducted Davis, California where the implementation of an on-street bicycle lane was to be evaluated [97]. Interviews were conducted before (*n* = 254) and after (*n* = 110) the bike lane was built, in which cyclists in households north of the implementation area were asked to draw their usual route to downtown or campus (which lay south of the area of interest). A number of other studies make use of similar interview techniques, but generally for intercepted cyclists in a specific study area [98,99].

Two Dutch studies investigate the implementation of a bicycle network scheme in Delft, one focussing on the route choices of cyclists prior to network implementation in 1982 [100], and the other discussing and comparing this with the detailed post-implementation data, collected three years later [101]. The pre-intervention data collection was performed in a single day, with 15 roadside locations where bicycle counts for bicycles were performed, together with interviews during which cyclists were given a mail-back route choice questionnaire [100]. In total, 60% or 2194 cyclists returned the route choice survey, whose main question concerned plotting the entirety of the journey undertaken on the day the interview. The second study, a project summary report, summarises the impacts of the initiatives in Delft. The study reports that “about 58% of the observed changes in bicycle volumes are caused by route shifts” but that “compared to travel time and directness, the type of facility is as such an unimportant route choice factor” [101].

Mail-back surveys were also given to passers-by in a study in Guelph, Canada [102] whilst another study by the same main author performed in Ottawa (*n* = 1603) and Toronto, Canada (*n* = 1360) used mail-back questionnaires attached to the parked bicycles [103]. Response rates in the second study were between 45% and 53% for the two cities, which is noteworthy considering the single contact and lack of follow up of participants. Another study used this approach in combination with manual distribution and collection in Phoenix, USA (*n* = 150) [104].

The third major category of recruitment in this section is for studies that require participants to fill in paper surveys on-site without the same follow up as in interviews. This was done at large events where booths could be established [105,106] or at workplaces or schools [107,108,109,110].

Finally, one study utilised a travel diary concept for the recording of routes as part of an ethnographic study of 26 cycling activists in Quito, Ecuador [111]. The participants in the study were asked to maintain a diary of incidents they had whilst cycling that were to be supplemented with freehand drawn mental maps (not assisted with a base map). Such an approach is highly dependent on the participants being very familiar with the area they cycle in and an ability to convey this information accurately in a sketch. Routes drawn in this manner may not have the same resolution as those that are map-assisted.

#### 3.4.2. Web-Based/Digitally Drawn Routes

The first study to use web-based or digitally drawn route choices was implemented amongst 142 staff and students of the University of Auckland [112]. Route choices of both cyclists and potential cyclists were recorded in a web-based GIS tool, although the exact details of the method are not explained.

The same concept was used in a second web-based data collection paper in the city of Copenhagen, for which 398 responses were received [113]. The study required cyclists to identify locations along their most recent route (which they traced turn-for-turn) where they had up to three positive and three negative experiences, leading to 890 points being located. Such experiences could have been the perception of danger at a blind corner or a positive comment regarding a widened bicycle lane. This data was collected in a Google Maps Application Programming Interface (API), allowing for data to be entered directly by users into a mapping interface with relative simplicity.

#### 3.4.3. Verbal/Written Descriptions 

Seven participant recalled route choice studies were located that use principally written or verbal descriptions of routes rather than visual depictions. The oldest study in this systematic literature review uses a described route choice data collection protocol in New York, USA. Two methods were devised, the first of which asked 35 families to list the route (presumably with road names) of their most recent bicycle trip and the second of which involved a verbal description of current route in 155 intercept interviews [114].

Three studies use even more limited descriptions of route choice, that only partially capture the route choice of cyclists—specifically through the inclusion of one to two additional points along a new piece of bicycle infrastructure in addition to origin and destination. The first of these evaluated the impact of a well-established off-road trail in Minneapolis, USA with 3121 cyclists stopped in a human intercept survey [115]. The second study using access and egress points to cycling infrastructure collected data via an online survey of usual bicycle trips in Montreal [116]. The study additionally asked for suggestions for new bicycle infrastructure locations. The high number of responses (*n* = 2917) was achieved through wide publication in conventional media formats, social networking websites and on the street. The third study uses only a single point (in addition to origin and destination) along a newly opened cycleway in Sydney to determine route choice; a point where 783 interviewed cyclists were intercepted as they waited at a traffic light [117]. Although cyclists were asked if they had changed route after the cycleway was opened, it was not required for them to specifically provide details of the existing route.

One approach to avoid poor geographical resolution is to interview cyclists about their route choice, where communication between interviewer and interviewee can ensure an accurate transcription of route choice details. This was done in a Vancouver telephone interview study with 74 participants for all modes, who had previously participated in a survey of cyclists and indicated willingness to be contacted for future research [118]. A benefit of this particular methodological set up was that participants had previously received a cycling map of Vancouver they were prompted to use as a visual aid when discussing typical routes with the interviewer. Route descriptions were also requested of 100 bike share users in Santiago, Chile, who were intercepted at stations by interviewers with paper-based surveys [119].

The poor ability of listing methods to accurately identify origin and destination mean that average trip lengths collected in this manner can be considerably shorter (or longer) than actual routes. Additionally many cyclists may not be sufficiently familiar with their environment to be able to give an accurate description of where they had or usually cycled. Benefits of this method are simplicity of execution, and in the case of interviews, the potential to gather richer open answers regarding variables that may have influenced route choice.

### 3.5. Accompanied Journey—Ride-Along and Tracking Based

#### 3.5.1. Ride-Along Survey

A technique with high potential for qualitative and ethnographic cycling research is the ride-along survey, a form of interview in which the interviewer accompanies the participant for part or their entire journey. Accompanied interviews were used to inform the design of a stated preference study from Transport for London [120]. In this case, ride-along surveys were performed with 16 participants, who were approached by interviewers at traffic lights and bike parks and then accompanied for 10–15 min of the remainder of their journey. A short roadside interview was conducted post-ride in which cyclists were asked questions about their route choice. Participants chose their routes themselves and were offered a gift voucher in return for their participation. The qualitative results here were used to inform the design of an online stated preference survey, which tested three key attributes: type of cycle lane, type of road and journey time.

The two remaining papers focussed upon more ethnographic research. The first of these incorporated ride-along interviews conducted with 15 inhabitants around Utrecht in The Netherlands, combining GPS tracking, tape-recording and video documentation (from interviewer perspective) [121]. Although GPS provides good location data, the video in this case provided information that was more important for the ethnographic study purpose (interaction with other road users during busy commuting times). This technique allowed researchers to retrace their ‘steps’ in visual field notes. Participants were recruited through an online discussion group and snowballing from contacts within the research group. Unlike the London study, the conducive environment to ‘conversational cycling’ in The Netherlands meant that the ride-along interview was indeed conducted mid-ride rather than post-ride, something the authors recognise may not be always be possible in other contexts.

The other ethnographic study also makes use of GPS, although in this case, was intended to examine the use of dedicated ride-logging smartphone applications [122]. Reflective diaries and structured interviews with 20 experienced club cyclists in and around Stoke-on-Trent, UK formed the basis of the research material whilst the author additionally accompanied the same cyclists on a number of group rides.

Benefits of the study design using ride-along surveys are the ability to interpret gestures and body language, together with other shared experiential factors that are not easily captured through the majority of other techniques. A downside can be that the observed behaviour of the cyclist (particularly risk-taking behaviour or the breaking of traffic laws) is influenced because they are conscious of being recorded or observed. This phenomenon can be mitigated if trust is developed between the interviewer and participant through more immersive researcher participation, as was demonstrated in an aforementioned study of cycling activists in Quito, Ecuador [111].

#### 3.5.2. Tracking

One study made use of tracking, but was GPS-assisted using devices fitted to trackers’ bicycles to allow for simpler data collection [123]. Methodologically the study involved tracking of 119 cyclists from a number of fixed destinations and a number of interception locations. A trip was considered finished after a cyclist dismounted. The route data for intercepted subjects is however incomplete since the initial part of the journey is not recorded. The research ethics of this methodology are not discussed in the article, although future studies should consider this.

### 3.6. Camera

Three studies make use of egocentric cameras as the primary means for determining route choice. Two used helmet-cameras [124,125] whilst one used two bicycle mounted cameras [126]. This naturalistic bicycle data was used to gather first hand cyclist experiences in the context of traffic safety and planning for cycling. The most recent of these used the video footage taken by 24 commuter cyclists in Plymouth, UK to perform video-guided interviews with the participant post-ride. The researcher reviewed the videos prior to the interview in order to develop a customised set of interview questions for each participant. Participants were also able to reflect upon what they saw in the video and volunteer comments. Neither of the other studies included this element, one of which was intended to capture overtaking distances on specific routes by the researcher themselves [126], the other classifying accidents or near-accidents [125].

Route choice was not explicitly a part of any of the studies, however all three studies have a forward-facing camera that shows the route being ridden. The video footage alone could be used to recreate route choices by a reviewer familiar with the area; however, those studies that were interested in both naturalistic behaviour and location tended to make use of cameras in GPS-instrumented bicycle set-ups as described in results Section 1 and Section 2.

### 3.7. Virtual Reality (VR) Simulated Environments

A VR cycling simulator called Cycle SPACES was developed in Breda in The Netherlands and is discussed in a pilot study of a proposed cycle superhighway in the same city [127]. A speed sensor allows users to adapt their speed in the virtual modelled environment (displayed with an Oculus Rift VR headset), whilst other variables such as time of day or the colour of the bicycle highway could be modified with push buttons on the handlebars. The participants were observed to react as expected in the different modelled environments, relaxing considerably in the future scenarios in which greater separation from traffic was displayed. Although route choices were not enabled in this experimental set-up, the addition of steering control together with eye-tracking, artificial intelligence of other road users, dynamic resistance and leaning have all been identified as future additions to the Cycle SPACES project.

A Japanese bicycle simulator experiment with 23 university students tests two scenarios that each provide the rider with visual feedback regarding speed, whilst a control group performs the experiment without visual input [128]. The experimental set up includes an exercise bicycle, speed sensor and large visual display. The first scenario shows a virtual bicycle icon on a Google map, which moves in accordance to pedalling speed, whilst the second scenario shows Google Street View images (taken at 10 m spacing) displayed in relation to pedalling speed. The route was fixed between the university and the nearest train station, without choice of route. Significant differences (*p* < 0.05) were found between the control and street image groups for enjoyment, outdoor-feeling and speed of cycling. Although route choice was not a studied element of the study, this would be a logical future step for virtual reality research.

Neither of the simulator-based VR studies study route choice unlike the majority of articles in this review, however as a relatively emergent field, the methods introduced here show promise for scaling up into this realm.

## 4. Discussion

**Figure 3:**
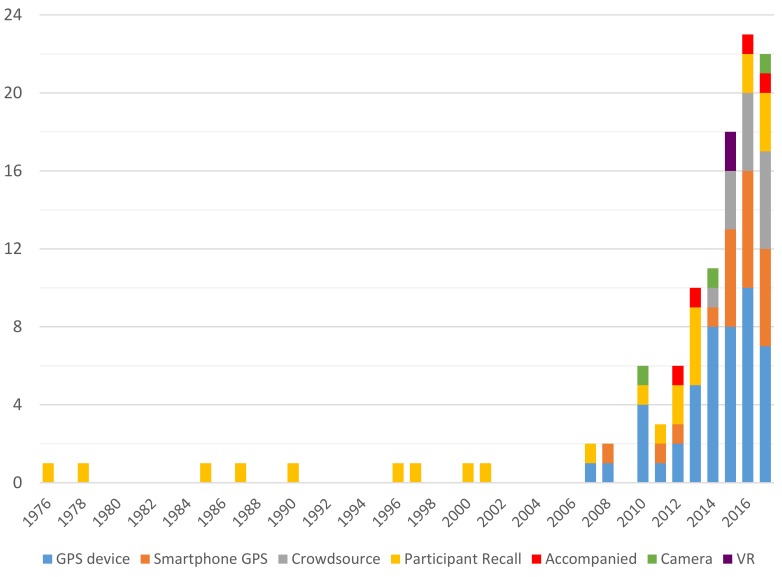
Revealed preference bicycle route choice publications for whole journeys (112) sorted by method family.

This study presents a systematic review of methods that have been applied to the study of whole journey bicycle movement. The research publications included within this review are mostly quite new, with 99 of the 112 studies published between 2010 and 2017. This is illustrated in Figure 3 below. In addition to the growth of research in general, much of the growth in research production can be attributed to the arrival of affordable GPS technology around the early-mid 2000s. GPS technology is used in over two thirds of the research papers collected through this systematic review, as illustrated in Figure 2 in the methods section. A modest increase in the number of papers utilising more mature methods like participant recalled routes can also be witnessed. Because the review required the digital availability of full-text articles, there is a systematic bias towards newer (especially post-internet) research, as is the case for the majority of review articles.

The numbers of participants is displayed in Figure 3 as a box-and-whisker plot for all 92 articles that reported this figure. Four method families are shown, whilst the remainder are aggregated under ‘other’ due to the low number of articles in the method families: accompanied trips, camera and virtual reality. The graph shows that the lowest numbers of participants does not vary greatly, but median number of participants can be quite different based on method family.

**Figure 4:**
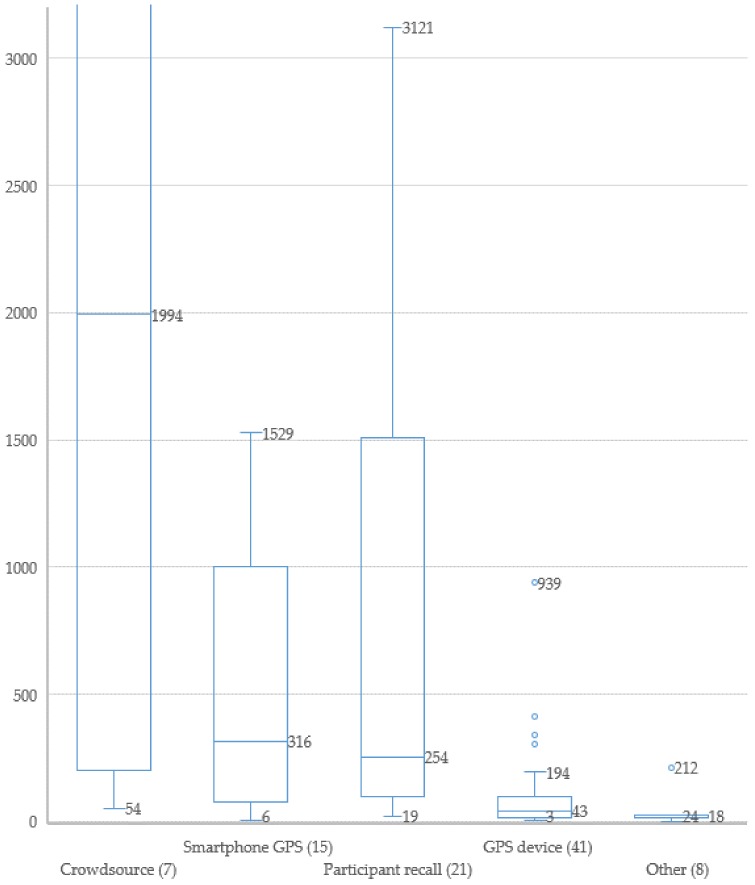
Reported number of participants by method family (number of included studies in parentheses). The category other includes accompanied trips, camera and VR.

Crowdsourcing studies have understandably the highest median number of participants (1994), although it should be noted that many of the studies reported findings from Strava, which do not presently provide individual route journeys. The high participant numbers in Strava are also the reason for not displaying the plot for crowdsourcing in its entirety (13,684 unique users are captured in the largest study covered by this review [86]). Smartphone GPS studies (316) and participant recalled route choice studies (254) follow next in median sample size, due most likely to the relative low-cost and simplicity of collecting data respectively. GPS devices, although utilising the same technology from crowdsourcing and smartphone studies, have a relatively lower median number of participants of 43, whilst the three remaining method families have a collective median of 18. This can be partly explained by each of the method families. Accompanied journey studies generally involved more qualitative methods (with generally lower sample sizes) making use of such techniques as interviews with participants. One notable exception involved tracking, unbeknown to 212 subjects [123]. Camera based studies are generally focussed on traffic safety, and whilst the data collection is not so time consuming as for interviews, requires prohibitively large amounts of time for manual data processing in order to establish route choices of more than a small number of participants. Machine learning and other image recognition technologies may assist in reducing the time required for this method in future work. The final method categorised under “other” in Figure 4 is virtual reality, which comprised only two studies, both at the demonstration stage. For such simulator studies, considerable amounts of time is required in creating the virtual environment to test, however after a model is created, it should be reasonably possible to sample much larger numbers of participants.

**Figure 5:**
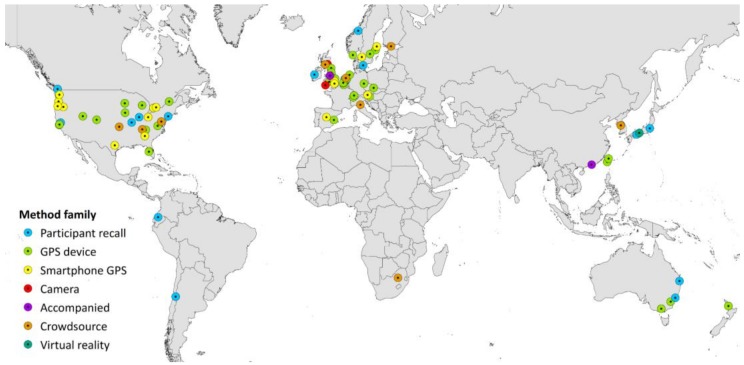
Geographical spread of the empirical research (112) by location of data collection.

The geographical spread of research according to city of data collection is shown in Figure 5. The USA is the most prolific producer of articles included in this review at 31 papers, followed by the UK and Canada with 12 and 11 respectively. Of the 112 empirical articles that form the basis of this review, 109 were performed in developed countries, defined here as those countries with a very high Human Development Index (HDI) in the 2016 Human Development Report (http://hdr.undp.org/en/countries). This means in practice that only 3% of the empirical data collected on whole-journey bicycle routes is in developing country contexts (China, Ecuador and South Africa). To put this in perspective, just over 20% of all research is produced by countries that do not have a very high HDI (based on submission institution of the ~33 million citable English language documents in Scopus from 1996 to 2016 http://www.scimagojr.com/countryrank.php). Hence, whilst over-representation of developed countries is present across all fields of research, it is considerably higher for bicycle route research. This is surprising given the relatively low costs of collecting route choice data (especially for surveys and smartphone apps) compared to research as a whole. However, this observation could indicate that funding is far from the sole determinant of research production. Indeed the low amount of cycling research may be due to a different focus in the research performed in developing countries, where the promotion of bicycles for transportation may be less prioritised.

### 4.1. Reasons for Route Choice

GPS is utilised in two thirds of all the research collected, providing a relatively high level of resolution for route choice behaviour between different streets and paths. However, with the exception of some smartphone studies and others with a user interface, the decision-making process behind the route choice is not typically revealed through this method. This is a common limitation of revealed preference studies, where the primary contribution is in showing the preference made rather than demonstrating why.

To understand more about the decisions being made by bicyclists, the other methods can be used. Follow-up interviews or surveys as part of GPS based studies has been demonstrated to provide attitudinal parameters [18,61,85]. Crowdsourcing as a method does not generally allow the study designer to ask questions of participants as the data is usually collected for a different purpose (sports applications for example), potentially some time ago. Participant recalled routes and accompanied journeys are typically performed as part of intercept surveys or organised interviews, which lend themselves well to establishing reasons for participants’ route choice. Cameras can be used to inform reasons for bicycle route choice, however in a limited manner since only visually identifiable reasons such as traffic or road surface condition in the immediate vicinity of the cyclist can be observed. The ideal situation is not only to accompany cyclists, but to record this on camera whilst interviewing cyclists, as was done in a qualitative study in Utrecht in the Netherlands [121]. The final method family of virtual reality allows testing of past, current or future scenarios, limited only by the time needed to create these virtual environments. Users can be interviewed whilst participating in the simulator environment, or can provide real time feedback through handlebar mounted buttons as discussed in Section 3.7 above. VR technologies are thus highly promising for participatory planning and user consultation of bicycle infrastructure, and it can be expected that this subset of the research will grow in coming years.

Revealed preference data alone does not however lack usefulness simply because it does not establish reasons for route choice. To the contrary, revealed preference data allows for large amounts of quantitative material to be collected, which combined with good sample representativeness, provides a holistic snapshot of a region’s cycling preferences. GPS based studies can sample many hundreds or even thousands of users, however combination with other methods such as cameras or follow-up interviews are necessary to begin to establish reasons for a particular route being chosen. Egocentric cameras alone can provide some of the context behind route choice, however data processing times for establishing a single route trace make this a more suitable supporting method to complement accompanied journeys or instrumented bicycle research designs. Surveys or interviews in which participants recall their routes can be highly informative across large samples, however generally only one to two routes can be recalled with reasonable accuracy. Short or infrequent trips, trip chains and trips taken some time ago are poorly represented in participant recalled route choice studies. Comprehensive travel diary studies that ask for route choice of all trips (together with the usual trip purpose and mode) are uncommon, potentially because of the effort of the recall is too great or the risk of incorrect routes being drawn too high. Such considerations should be made in the context of the target sample and research purpose. If the sample is young children, GPS based tracking may prove to be difficult because of increased privacy concerns from parents and research ethics committees. Likewise, if the purpose of the study were to quantitatively determine the surface riding quality, the use of accelerometers would be more useful than interviews.

### 4.2. Representation

An issue that was witnessed repeatedly across different methods is the statistical representation of the participants. One study summarises the typical representation problems that occur in revealed preference bicycle studies: “the GPS participants were slightly older, were more likely to have a college degree, had higher incomes, and were more likely to have full-time jobs than other regular cyclists” [20]. Similar results were found by other studies, despite large numbers of participants [93].

In many cases the research aims target particular groups of participants, such as through the specific targeting of elderly bike users [37,42]. The intention here is evidently not to assume representativeness by choosing one demographic, however other studies also chose specific users to the detriment of representation. The best example of this is the adult commuter cyclist target group, who are frequently chosen because they are simplest to recruit, although not necessarily most numerous or representative of the general population [15,18,21,39,46,58,62,99,102,103,119,123,124,128,129]. Unless the sample is targeted with the specific aim to improve representativeness, we risk producing results that poorly demonstrate how bicyclists behave, let alone the target cycling population (those who do not presently use a bicycle regularly).

A number of studies specifically avoided targeting a particular transport mode during recruitment in order to get a better picture of normal road user behaviour [61,72,73,96]. It should be noted however that the lack of representation amongst cyclists targeted for GPS studies is not necessarily improved when considering all transport modes. One combined GPS and travel diary study reported participants were better educated, wealthier and older than the those captured by census data; similar attributes for the samples in much of the bicycle research [61].

Adult men are highly overrepresented in many sports and recreation-focussed smartphone apps. In some low cycling areas, this may indeed be representative of the cycling population, however the consideration should also be made for the main target group of bicycle promotion initiatives: infrequent and non-cyclists. This is in contrast to most GPS data studies, which seek to achieve better gender and age balance during participant recruitment [12].

Removing technology-based data collection methods from the discussion gives a different perspective. A large study of cyclists conducted in Groningen, The Netherlands (*n* = 1012) and Växjö, Sweden (*n* = 1003) stopped participants mid-journey to answer questions concerning trip purpose, origin-destination, actual route choice and route choice motive [98]. The random sampling process meant that both gender and age were well represented, although the authors explain that the sampling sites were selected based on high vulnerable road user flows, and were thus not reflective of each city’s general population.

The sampling methodology, as much as the actual data collection method can determine the representativeness of the sample. In the example above, random sampling was used together with a survey requiring little participant involvement—resulting in excellent representation for the area sampled. If the sampling methodology involves searching for potential participants on bicycle forums, it should be considered that the forums are not representative of the general cycling public, but rather a special-interest subgroup of cyclists. Similarly, the use of sports applications as a data collection method predetermines the sample to be biased towards middle-aged men. Good representation is not simple to achieve, but the targeting of everyday cyclists is best achieved through random sampling in the area of interest. This can mean more time is required to achieve the desired sample size, but can also be performed using randomised telephone directory lists or mail-out surveys.

### 4.3. Future Research

Aerial drone and computer vision software (that automatically identifies mode, position and velocity of multiple road users) is being increasingly used in combination to record traffic behaviour. Presently this has been applied mostly to intersection or individual segments of interest, due to a limited scope in a single camera.

Although elements of route choice can be easily observed in this manner, the capture of entire route choices of cyclists is unlikely to be possible unless either the drone tracks individuals or multiple elevated cameras are used to identify and subsequently re-identify the same users. This is prohibitively difficult in a manual video analysis study; however, advanced computer vision technology could allow the identification and subsequent re-identification at multiple points in a network of fixed elevated cameras. Surveillance and privacy related issues may however make both of these methods unworkable from a research ethics standpoint, and must be seriously considered prior to implementation.

## 5. Conclusions

This literature review provides an overview of the techniques that are available to track whole-journey cyclist movements in the bicycle network and by doing so can allow for insights concerning the preferences of bicycle users. Sample selection must however be taken into consideration concerning the transferability of such insights. Samples should ideally be broadly representative of the general public or preferably the target audience (for example young children if the aim is to promote cycling to school). The prioritisation of infrastructure spending in accordance with what is demonstrated to have the greatest impact for a representative sample will most efficiently allow for an increase in cycling modal share.

The literature review revealed some key findings regarding the collection of cyclist data. GPS-related data sources were used in two thirds of the empirical studies covered by this review. Experimental research designs using GPS-instrumented bicycle setups were common in the literature, however there appears to be more growth potential in the use of smartphone GPS and crowdsourcing. For these data sources, automatic mode-classification algorithms are often required. Less technology-oriented approaches involving participant recall of routes are demonstrated to be able to achieve similar levels of participation to smartphone-based approaches, although usually restricted to a single route. GPS alone does not provide the resolution necessary to determine the choice of infrastructure located on a single segment (such as footpath or bicycle lane), and in these cases camera technology, interviews or accompanied journeys is necessary.

Data privacy was at times observed to hinder the participant recruitment process, especially when considering passive smartphone recorded data. The availability of location data from smartphones can reveal a significant amount of personal information including work, home, leisure activities and behaviour. It is thus essential that any proposed data collection approach ensures participant anonymity and is approved by a local research ethics committee prior to implementation. This includes not only personal details but also the obscuration of precise origins and destinations if these could be used to identify participants. Participants in travel surveys of this nature must also be well informed of the process by which the research is conducted.

Finally crowdsourcing-based studies, together with virtual reality simulators and advanced computer vision processing of video data can be expected to develop significantly in the coming years, providing new and rich future data sources for the study of bicycle route choice.

## Appendix A

**Table A1:** Classification of the 112 empirical studies based on principal route choice methods used into family and primary method group.

Family	Sub-Group	Main Benefits	Main Drawbacks	Integration with Other Methods	Sources
GPS device—not including smartphone GPS (47)	Instrumented research bicycle	Pre-configured for data collection Flexible experimental set up Consistent data (same bicycle for all users)	Participants must charge instrumentation for longer term studies	√√√	[17,25,26,30,32,33,34,35,36]
	Instrumented research pedal-assisted electric bicycle (pedelec)	Same as for instrumented research bicycle Pedelec power source allows longer term data collection Simple activation of GPS on start up	Initial purchase cost of bicycle Unfamiliarity of users with pedelecs can result in abnormal cycling behaviour	√√√	[16,22,24,28,37]
	Instrumented participant bicycle	Familiarity with own bicycle gives good naturalistic data	Difficulty of comparing secondary data sources such as vibration because of bicycle variety	√√	[27,31,38,39,40,41]
	Instrumented participant pedelec	Pedelec power source allows longer term data collection Simple activation of GPS on start up	Difficulty in recruiting participants (if low numbers of pedelecs used)	√√√	[15,42]
	Instrumented quasi-bikeshare (university pilot scheme)	Comparison of many users possible with autonomous data retrieval	Maintenance and purchase cost of pilot scheme	√√	[23,43]
	Instrumented participant (two or more devices borne by participant)	Intermodal Pre-configured for data collection User interface (if present) can assist engagement with user (for travel survey or to remember charging)	Mode identification necessary Can be troublesome for users to carry all instrumentation (depending on size)	√√	[14,19,46,47,48]
	GPS integrated in other device (excl. smartphone and accelerometer)	Intermodal User interface (if present) can assist engagement with user (for travel survey or to remember charging) Compact device, easy to carry in pocket or bag	Mode identification necessary	√	[20,49,50,51]
	Wearable GPS device	Intermodal Low participant burden	Mode identification necessary Participants can forget to charge battery	√	[52,53,54,55,56]
	Participant-borne GPS device (no mention of wearability)	Intermodal Compact device, easy to carry in pocket or bag	Mode identification necessary Participants can forget to take GPS on all journeys or to charge battery	√	[18,21,29,57,58,59,60,61]
	Handlebar-mounted GPS participant bicycle (without additional instrumentation)	No need to perform mode identification Participant does not need to remember to take GPS on bicycle journeys	Participants can easily forget to charge battery if taking infrequent bicycle trips	√	[62]
Smartphone GPS—participants solicited for research (20)	Passive user registration—no input required from user, trips detected automatically	Intermodal Can be combined with travel diary (participants can confirm mode/trip purpose)	Mode identification necessary Recruitment more difficult than for active user apps, where participant has more control over the data they record	√√	[66,72,73]
	Passive user registration—smartphone based instrumented research bicycle (and pedelec)	Same as for GPS -instrumented research bicycle/pedelec Simpler configuration of instrumentation with smartphone (if only basic sensors used)	Same as for GPS -instrumented research bicycle/pedelec Energy saving required, which reduces data quality	√√√	[67,68,70,74]
	Active user registration (user activates GPS through app)—using or developed from the app “CycleTracks”	Few battery problems Proven method—readily obtainable for other studies User control over data provision (can opt to not record specific journeys)	User must engage with trip to begin, pause and stop recording	√√	[69,75,76,77,78,79,80]
	Active user registration—other customised app specifically designed to provide data for planners/researchers	Backend system allows simple access to necessary user data User control over data provision	User must engage with trip to begin, pause and stop recording App must be developed	√√	[81,82,83,84,85]
	Active user registration—via recreation-oriented apps	Collating of data can be automated via access tokens No need to develop app User control over data provision	User must engage with trip to begin, pause and stop recording User must upload data in some experimental designs	√	[71]
Crowdsourcing—data generally not solicited by researchers (13)	Recreational/sports application (smartphone)—data collection not solicited by researchers	Data obtained en masse via some application owners Longitudinal data Detailed data can be volunteered	Sports app users are not representative of general public Data privacy often restricts full access to individual trips	√	[86,87,88,89,90,91]
	Customised smartphone application for nationwide data collection (intended for data sharing)	Large dataset Whole country data for comparison	Data privacy often restricts full access to individual trips	√√	[92,93,94]
	Volunteered data from previously collected routes (access to existing data solicited by researchers)	Same as for sports applications, but only for volunteered routes User control over data provision	Representativeness Recruitment of GPS users can be difficult	√	[95]
	Instrumented public bikeshare bicycles	Representativeness Large dataset Longitudinal access can be obtained via operators of GPS-enabled bikeshare fleet	Generally limited to GPS only User data may be retained for data privacy reasons	√√	[44,45]
	Citizen science crowdsourcing platform (Amazon Mechanical Turk)—for smartphone study	Affordable means of obtaining responses with low researcher burden Can sample from global network of users Cannot meet with participants readily	Difficult to be geographically specific (platform users are spread globally)	√√	[96]
Participant recalled route—hand-drawn (15), web-based (2) or verbal/written description (6)	Hand drawn from interview of participants	Interviewer can ask for clarification (including use of different parts of street) as route is drawn	Accuracy—lack of familiarity with area Limited application beyond usual journey or most recent	√√	[97,98,99]
	Hand drawn from mail-back surveys after interception of passers-by	Large numbers of respondents possible Interception not always practical or legal (some jurisdictions classify cyclists in a similar manner to motorised vehicle users)	Relies on map familiarity Limited application beyond usual journey or most recent	√	[100,101,102,103]
	Hand drawn from paper surveys completed on-site (at destination)	Interviewers can assist as required Ease of recruiting large numbers of participants Can tailor map according to destination environment (such as workplace)	Relies on map familiarity Limited application beyond usual journey or most recent	√	[105,106,107,108,109,110]
	Hand drawn from printable survey distributed online/advertisements	Large survey distribution possible (through forums, newspapers, noticeboards, bicycle shops etc.)	Relies on map familiarity Limited application beyond usual journey or most recent High participant burden to print survey Map detail vs. size trade-off (whole city is difficult to appropriately represent in map)	√	[104]
	Hand drawn from mental mapping journal (specific target audience)	Longitudinal time frame (multiple journeys, not just most recent or usual) Participant reflection in journal	No map guidance Uncertainty in matching participant-recalled routes to reality	√	[111]
	Mapping Application Programming Interface (API) for online route tracing	Low participant burden—large quantitative datasets possible Routing algorithms can be used to suggest routes for origin-destination Can be integrated with Public Participation Geographic Information System (PPGIS) for obtaining geo-located user feedback	Only limited numbers of routes generally recalled Relies on map familiarity	√√	[112,113]
	Description using list of road names used	Quick methodology, readily applicable to intercept interviews	Accuracy—recall error/lack of familiarity with road names	√	[114]
	Description using points/landmarks along route (verbal or online)	Quick methodology, readily applicable to intercept interviews	Imprecise—often only two points along route	√	[115,116,117]
	Description from detailed interview	Interviewer can ask for clarification Can be map-assisted	Accuracy—lack of familiarity with area	√√	[118,119]
Accompanied trip—ride-along (3) or tracking (1)	Ride-along	Experiential ‘sensescapes’ can be captured and discussed real-time Best captures the context of the bicycle user	Transcribing can be difficult (reduced clarity from participants when cycling in addition to noise)	√√√	[121,122]
	Post ride-along interview	Reasons for choices can be explained Suitable in environments where conversational cycling not practical	Lack of real-time participant reflection compared to ride-along	√√√	[120]
	Unobtrusive tracking from distance	Recruitment not necessary	Lack of ‘participant’ consent Time intensive Difficult to capture journeys originating from homes	√	[123]
Camera (3)	Egocentric camera on rider/bicycle (commonly used as a supplementary method)	Naturalistic behaviour and interactions can be captured including traffic violations and interactions with other road users	Time intensive for route choice Requires researcher familiarity with area	√√	[124,125,126]
Virtual Reality (VR) (2)	Immersion in virtual/photographed street environment	Proposed changes to built environment can be evaluated in detail Non-cyclists’ revealed preference behaviour can be tested in a safe environment	Modelling time requirement for virtual environment Some participants experience dizziness in VR simulators	√√	[127,128]

**Table A2:** Hand-drawn route recall studies.

Reference	Main Field of Application	Respondents (Valid)	Type of Route Drawn	Recruitment Methods
Lott et al., 1978 [97]	Transport planning—before and after evaluation	364 cyclists in Davis, USA	Usual route to downtown or campus (located on the other side of infrastructure)	Interviews of cyclists at their homes before and after the infrastructure development.
Van Maarseveen et al., 1985 [100]	Transport planning—before evaluation	2194 cyclists in Delft, The Netherlands	Current journey when intercepted	Distributed mail-back survey to intercepted cyclists (60% return rate)
Wilmink & Hartman, 1987 [101]	Transport planning—before and after network evaluation	Cyclists in Delft, The Netherlands	Current journey when intercepted	Distributed mail-back survey to intercepted cyclists (return rate not stated)
Van Schagen, 1990 [98]	Data collection to guide creation of a route choice model	Cyclists in Groningen, The Netherlands (1012) and Växjö, Sweden (1003)	Current journey when intercepted	Intercept interview in areas with high cyclist numbers
Aultman-Hall, 1996 [103]	Safety—bicycle routes and accident locations	Bicycle commuters in Ottawa (1603) and Toronto, Canada (1360)	Current regular bicycle commute	Mail-back questionnaires attached to the cross-bars of parked bicycles (49% returned)
Aultman-Hall et al., 1997 [102]	Transport planning—GIS demonstration	338 cyclists in Guelph, Canada	Commonly used bicycle journeys	Mail out survey (10% return), distributed mail-back survey to intercepted cyclists and through placement in bicycle shops
Hyodo et al., 2000 [108]	Route choice model—railway station accessibility	Cyclists in Utsunomiya (502) and Kurume, Japan (252)	Usual bike routes to school or work	Distributed surveys at high schools, bicycle parking facilities and railway stations
Howard & Burns, 2001 [104]	Transport planning—level of stress	150 experienced commuter cyclists in Phoenix, USA	Last journey to work, not map-assisted	Printable survey distributed digitally through webpages, email, cycling list server, and advertised/physically distributed at bike shops/community meetings
Suzuki et al., 2012 [109]	Transport planning—traffic volume and bicycle network issues	1419 cyclists in Takamatsu, Japan	Written detailed route on map	Paper survey sent to offices and high schools, interviews in downtown areas
Manum & Nordström, 2013 [107]	Transport planning—bikeability	Commuter cyclists in Trondheim, Norway	Route between home and workplace	Paper survey distributed at three employer hubs in city centre
Kang & Fricker, 2013 [99]	Transport planning—on-street vs. off-street facility use	178 cyclists in West Lafayette, USA	Route to university indicated visually on map (to interviewer)	Intercept interviews at bicycle racks at destination
Yang & Mesbah, 2013 [110]	Stated vs. revealed preference for factors influencing cycling	19 cyclists in Brisbane, Australia	Last cycling trip ending at home	Pilot study amongst university students and staff
Manton et al., 2016 [106]	Safety—cycling risk (also using stated preference)	104 cyclists in Galway, Ireland	Regularly used cycling routes, colour-coded according to perceived risk	Paper based surveys completed at large university events
Boettge et al., 2017 [105]	Transport planning—cycling risk assessment for siting facilities	89 cyclists in St. Louis, USA	Recently used bicycle route, colour-coded according to perceived risk	Booths at cycling events allowing participants to complete survey on site
Gamble, Snizek, & Nielsen, 2017 [111]	Ethnography, understanding cycling activism	26 cyclists in Quito, Ecuador	Freehand mental map of noteworthy recent journeys in journal (not map assisted)	Specific target audience of cycling activists—who received a disposable camera and journal with prompts

## References

[1] Buehler R., Pucher J. (2011). Sustainable Transport in Freiburg: Lessons from Germany’s Environmental Capital. Int. J. Sustain. Transp..

[2] Macmillan A., Connor J., Witten K., Kearns R., Rees D., Woodward A. (2014). The Societal Costs and Benefits of Commuter Bicycling: Simulating the Effects of Specific Policies Using System Dynamics Modeling. Environ. Health Perspect..

[3] De Hartog J.J., Boogaard H., Nijland H., Hoek G. (2010). Do the health benefits of cycling outweigh the risks?. Environ. Health Perspect..

[4] Ryus P., Ferguson E., Laustsen K.M., Schneider R.J., Proulx F.R., Hull T., Miranda-Moreno L. (2014). NCHRP Web-Only Document 205: Methods and Technologies for Pedestrian and Bicycle Volume Data Collection.

[5] Ryus P., Butsick A., Proulx F.R., Schneider R.J., Hull T. (2017). NCHRP Web-Only Document 229: Methods and Technologies for Pedestrian and Bicycle Volume Data Collection: Phase 2.

[6] Nordback K.L. (2012). Estimating Annual Average Daily Bicyclists and Analyzing Cyclist Safety at Urban Intersections,. Ph.D. Thesis.

[7] Minge E., Falero C., Lindsey G., Petesch M., Vorvick T. (2017). Bicycle and Pedestrian Data Collection Manual. Report MN/RC 2017-03.

[8] Kothuri S., Nordback K., Schrope A., Phillips T., Figliozzi M. (2017). Bicycle and Pedestrian Counts at Signalized Intersections Using Existing Infrastructure. Transp. Res. Rec. J. Transp. Res. Board.

[9] Krenn P.J., Titze S., Oja P., Jones A., Ogilvie D. (2011). Use of global positioning systems to study physical activity and the environment: A systematic review. Am. J. Prev. Med..

[10] Loveday A., Sherar L.B., Sanders J.P., Sanderson P.W., Esliger D.W. (2015). Technologies that assess the location of physical activity and sedentary behavior: A systematic review. J. Med. Internet Res..

[11] Buehler R., Dill J. (2015). Bikeway Networks: A Review of Effects on Cycling. Transp. Rev..

[12] Romanillos G., Zaltz Austwick M., Ettema D., De Kruijf J. (2016). Big Data and Cycling. Transp. Rev..

[13] Moher D., Liberati A., Tetzlaff J., Altman D.G., Altman D., Antes G., Atkins D., Barbour V., Barrowman N., Berlin J.A. (2009). Preferred reporting items for systematic reviews and meta-analyses: The PRISMA statement. PLoS Med..

[14] Lindsey G., Hankey S., Wang X., Chen J., Gorjestani A. (2013). Feasibility of Using GPS to Track Bicycle Lane Positioning. Report CTS 13-16.

[15] Lopez A.J., Astegiano P., Gautama S., Ochoa D., Tampère C., Beckx C. (2017). Unveiling E-Bike Potential for Commuting Trips from GPS Traces. ISPRS Int. J. Geo-Informat..

[16] Dozza M., Bianchi Piccinini G.F., Werneke J. (2016). Using naturalistic data to assess e-cyclist behavior. Transp. Res. Part F Traffic Psychol. Behav..

[17] Garcia A., Gomez F.A., Llorca C., Angel-Domenech A. (2015). Effect of width and boundary conditions on meeting maneuvers on two-way separated cycle tracks. Accid. Anal. Prev..

[18] Plazier P.A., Weitkamp G., Berg A.E. (2017). Van Den “Cycling was never so easy!” An analysis of e-bike commuters’ motives, travel behaviour and experiences using GPS-tracking and interviews. J. Transp. Geogr..

[19] Pooley C., Whyatt D., Walker M., Davies G., Coulton P., Bamford W. (2010). Understanding the school journey: Integrating data on travel and environment. Environ. Plan. A.

[20] Dill J., Gliebe J. (2008). Understanding and Measuring Bicycling Behavior: A Focus on Travel Time and Route Choice. Report OTREC-RR-08-03.

[21] Harvey F., Krizek K. (2007). Commuter Bicyclist Behavior and Facility Disruption. Report MN/RC-2007-15.

[22] Paefgen J., Michahelles F., Boll s., Schöning J. (2010). Inferring usage characteristics of electric bicycles from position information. Proceedings of the 3rd International Workshop on Location and the Web—LocWeb ’10;.

[23] Ioakimidis C.S., Rycerski P., Koutra S., Genikomsakis K.N. (2016). A university e-bike sharing system used as a real-time monitoring emissions tool under a smart city concept. EVS29 International Battery, Hybrid and Fuel Cell Electric Vehicle Symposium.

[24] Allemann D., Raubal M., Bacao F., Santos M.Y., Painho M. (2015). Usage Differences between Bikes and E-Bikes. AGILE 2015, Geographic Information Science as an Enabler of Smarter Cities and Communities.

[25] Oh J., Kwigizile V., Ro K., Feizi A., Kostich W., Abdullah R., Hasan H., Mousa F.A. (2017). Effect of Cycling Skills on Bicycle Safety and Comfort Associated with Bicycle Infrastructure and Environment.

[26] Lai C.H., Doong J.L., Chuang K.H., Hsu C.C. (2015). The Likelihoods of Bicyclist Steering Patterns in Response to Being Passed by Motorists. Procedia Manuf..

[27] Kircher K., Ahlstrom C., Palmqvist L., Adell E. (2015). Bicyclists’ speed adaptation strategies when conducting self-paced vs. system-paced smartphone tasks in traffic. Transp. Res. Part F Traffic Psychol. Behav..

[28] Berntsen S., Malnes L., Langåker A., Bere E. (2017). Physical activity when riding an electric assisted bicycle. Int. J. Behav. Nutr. Phys. Act..

[29] Shin D.K. (2016). Explanation of Factors Influencing Cyclists’ Route Choice Using Actual Route Data from Cyclists. Ph.D. Thesis.

[30] Jahangiri A., Elhenawy M., Rakha H., Dingus T.A. (2016). Investigating cyclist violations at signal-controlled intersections using naturalistic cycling data. Proceedings of the 2016 IEEE 19th International Conference on Intelligent Transportation Systems (ITSC).

[31] Lin P.-S., Kourtellis A., Katkoori S., Chen C., Cruse L. (2017). Naturalistic Bicycling Behavior Pilot Study. Report BDV25-977-13.

[32] Chuang K.H., Hsu C.C., Lai C.H., Doong J.L., Jeng M.C. (2013). The use of a quasi-naturalistic riding method to investigate bicyclists’ behaviors when motorists pass. Accid. Anal. Prev..

[33] Jarjour S., Jerrett M., Westerdahl D., de Nazelle A., Hanning C., Daly L., Lipsitt J., Balmes J. (2013). Cyclist route choice, traffic-related air pollution, and lung function: A scripted exposure study. Environ. Health.

[34] Dozza M., Fernandez A. (2014). Understanding bicycle dynamics and cyclist behavior from naturalistic field data (November 2012). IEEE Trans. Intell. Transp. Syst..

[35] Bigazzi A.Y., Figliozzi M.A. (2015). Roadway determinants of bicyclist exposure to volatile organic compounds and carbon monoxide. Transp. Res. Part D Transp. Environ..

[36] Etemad H., Costello S.B., Wilson D.J. (2016). Using an instrumented bicycle to help understand cyclists’ perception of risk. IPENZ Transport Group Conference.

[37] Kovácsová N., de Winter J.C.F., Schwab A.L., Christoph M., Twisk D.A.M., Hagenzieker M.P. (2016). Riding performance on a conventional bicycle and a pedelec in low speed exercises: Objective and subjective evaluation of middle-aged and older persons. Transp. Res. Part F Traffic Psychol. Behav..

[38] Cole-Hunter T.A. (2012). Effects of Air Pollution Exposure on Adult Bicycle Commuters: An Investigation of Respiratory Health, Motorised Traffic Proximity and the Utility of Commute Re-Routing,. Ph.D. Thesis.

[39] Gustafsson L., Archer J. (2013). A naturalistic study of commuter cyclists in the greater Stockholm area. Accid. Anal. Prev..

[40] Bíl M., Andrášik R., Kubeček J. (2015). How comfortable are your cycling tracks? A new method for objective bicycle vibration measurement. Transp. Res. Part C Emerg. Technol..

[41] Parkin J., Rotheram J. (2010). Design speeds and acceleration characteristics of bicycle traffic for use in planning, design and appraisal. Transp. Policy.

[42] Gelhert T. (2014). Traffic Safety of Electric Bicycles.

[43] Langford B.C., Chen J., Cherry C.R. (2015). Risky riding: Naturalistic methods comparing safety behavior from conventional bicycle riders and electric bike riders. Accid. Anal. Prev..

[44] Wergin J., Buehler R. (2017). Where Do Bikeshare Bikes Actually Go?. Transp. Res. Rec. J. Transp. Res. Board.

[45] Khatri R., Cherry C.R., Nambisan S.S., Han L.D. (2016). Modeling Route Choice of Utilitarian Bikeshare Users with GPS Data. Transp. Res. Rec. J. Transp. Res. Board.

[46] Good N., Mölter A., Ackerson C., Bachand A., Carpenter T., Clark M.L., Fedak K.M., Kayne A., Koehler K., Moore B. (2016). The Fort Collins Commuter Study: Impact of route type and transport mode on personal exposure to multiple air pollutants. J. Expo. Sci. Environ. Epidemiol..

[47] Dill J., McNeil N., Broach J., Ma L. (2014). Bicycle boulevards and changes in physical activity and active transportation: Findings from a natural experiment. Prev. Med..

[48] Brown B.B., Tharp D., Tribby C.P., Smith K.R., Miller H.J., Werner C.M. (2016). Changes in bicycling over time associated with a new bike lane: Relations with kilocalories energy expenditure and body mass index. J. Transp. Health.

[49] Johnson M., Chong D., Carroll J., Katz R., Oxley J., Charlton J. (2014). Naturalistic Cycling Study: Identifying Risk Factors for Cyclists in the Australian Capital Territory. Report 322.

[50] Hamann C.J., Pooley M., McGehee D., Peek-Asa C. A naturalistic study of child and adult bicycling behaviours and risk exposure. Proceedings of the International Cycling Safety Conference;.

[51] Hatfield J., Dozza M., Patton D.A., Maharaj P., Boufous S., Eveston T. (2017). On the use of naturalistic methods to examine safety-relevant behaviours amongst children and evaluate a cycling education program. Accid. Anal. Prev..

[52] Casello J.M., Rewa K.C., Nour A. An Analysis of Empirical Evidence of Cyclists’ Route Choice and Its Implications for Planning. Proceedings of the Transportation Research Board 91st Annual Meeting.

[53] Casello J.M., Nour A., Rewa K.C., Hill J. Analysis of Stated-Preference and GPS Data for Bicycle Travel Forecasting. Proceedings of the Transportation Research Board 90th Annual Meeting.

[54] Wati K., Burke M., Sipe N., Dodson J. (2013). Children’s cycling trends, accessibility to and utilisation of urban facilities in selected Australian urban environments. Australasian Transport Research Forum.

[55] Krenn P.J., Oja P., Titze S. (2014). Route choices of transport bicyclists: A comparison of actually used and shortest routes. Int. J. Behav. Nutr. Phys. Act..

[56] Dessing D., de Vries S.I., Hegeman G., Verhagen E., van Mechelen W., Pierik F.H. (2016). Children’s route choice during active transportation to school: difference between shortest and actual route. Int. J. Behav. Nutr. Phys. Act..

[57] Menghini G., Carrasco N., Schüssler N., Axhausen K.W. (2010). Route choice of cyclists in Zurich. Transp. Res. Part A Policy Pract..

[58] Dalton A.M., Jones A.P., Panter J., Ogilvie D. (2014). Are GIS-modelled routes a useful proxy for the actual routes followed by commuters?. J. Transp. Health.

[59] Yeboah G., Alvanides S., Jokar Arsanjani J., Zipf A., Mooney P., Helbich M. (2015). Route Choice Analysis of Urban Cycling Behaviors Using OpenStreetMap: Evidence from a British Urban Environment. OpenStreetMap in GIScience.

[60] Apparicio P., Carrier M., Gelb J., Séguin A.M., Kingham S. (2016). Cyclists’ exposure to air pollution and road traffic noise in central city neighbourhoods of Montreal. J. Transp. Geogr..

[61] Montini L., Antoniou C., Axhausen K.W. Route and Mode Choice Models Using GPS Data. Proceedings of the Transportation Research Board 96th Annual Meeting.

[62] Luo D., Ma X. Analysis of Cyclist Behavior Using Naturalistic Data: Data Processing for Model Development. Proceedings of the 3rd International Cycling Safety Conference.

[63] Wolf J., Guensler R., Bachman W. (2001). Elimination of the Travel Diary: Experiment to Derive Trip Purpose from Global Positioning System Travel Data. Transp. Res. Rec. J. Transp. Res. Board.

[64] Wolf J. Application of New Technologies in Travel Surveys. Proceedings of the International Conference on Transport Survey Quality and Innovation.

[65] Doherty S.T., Noël N., Gosselin M.-L., Sirois C., Ueno M., Murakami E., Pisarski A.E. (1999). Moving Beyond Observed Outcomes: Integrating Global Positioning Systems and Interactive Computer-Based Travel Behavior Surveys. TRB Transportation Research Circular—Personal Travel. The Long and Short of It.

[66] Sandsjö L., Sjöqvist B.A., Candefjord S. A Concept for Naturalistic Data Collection for Vulnerable Road Users Using a Smartphone-Based Platform. Proceedings of the International Technical Conference on the Enhanced Safety of Vehicles (ESV).

[67] Kiefer C., Behrendt F. (2016). Smart e-bike monitoring system: real-time open source and open hardware GPS assistance and sensor data for electrically-assisted bicycles. IET Intell. Transp. Syst..

[68] Liu F., Figliozzi M., Caviedes A., Le H., Mai L. (2016). Utilizing Egocentric Video and Sensors to Conduct Naturalistic Bicycling Studies. Report NITC-RR-805.

[69] Watkins K.E., LeDantec C. (2016). Using Crowdsourcing to Prioritize Bicycle Route Network Improvements. Report FHWA-GA-16-1439.

[70] Rios I., Golab L., Keshav S. (2016). Analyzing the usage patterns of electric bicycles. Proceedings of the Workshop on Electric Vehicle Systems, Data, and Applications—EV-SYS ’16.

[71] Romanillos G., Zaltz Austwick M. (2016). Madrid cycle track: Visualizing the cyclable city. J. Maps.

[72] Gonzalez P.A., Weinstein J.S., Barbeau S.J., Labrador M.A., Winters P.L., Georggi N.L., Perez R.A. Automating Mode Detection Using Neural Networks and Assisted GPS Data Collected Using GPS-Enabled Mobile Phones. Proceedings of the 15th World Congress on Intelligent Transport Systems.

[73] Nour A., Casello J.M., Hellinga B. Developing and Optimizing a Transportation Mode Inference Model Utilizing Data from GPS Embedded Smartphones. Proceedings of the Transportation Research Board 94th Annual Meeting.

[74] Saleh P., Otte D., Krettek C. (2014). E-bicycles—Bicycles or mopeds? Overview about the project “SEEKING—SAFE E-BIKING.” In International Conference on ESAR “Expert Symposium on Accident Research”.

[75] Charlton B., Sall E., Schwartz M., Hood J. Bicycle Route Choice Data Collection using GPS-Enabled Smartphones. Proceedings of The Transportation Research Board 90th Annual Meeting.

[76] Hudson J.G., Duthie J.C., Rathod Y.K., Larsen K.A., Meyer J.L. (2012). Using Smartphones to Collect Bicycle Travel Data in Texas. DOT Grant No. DTRT06-G-0044.

[77] Chen P., Shen Q., Childress S. (2017). A GPS data-based analysis of built environment influences on bicyclist route preferences. Int. J. Sustain. Transp..

[78] Akar G., Park Y. (2017). Tracking Bicyclists’ Route Choices, Case Study: The Ohio State University. NEXTRANS Project No. 171OSUY2.2.

[79] Figliozzi M.A., Blanc B.P. (2015). Evaluating the Use of Crowdsourcing as a Data Collection Method for Bicycle Performance Measures and Identification of Facility Improvement Needs. Report SPR 768.

[80] Zimmermann M., Mai T., Frejinger E. (2017). Bike route choice modeling using GPS data without choice sets of paths. Transp. Res. Part C Emerg. Technol..

[81] Li S., Muresan M., Fu L. Cycling in Toronto: Route Choice Behaviour and Implications to Infrastructure Planning. Proceedings of the Transportation Research Board 96th Annual Meeting.

[82] Strauss J., Miranda-Moreno L.F. (2017). Speed, travel time and delay for intersections and road segments in the Montreal network using cyclist Smartphone GPS data. Transp. Res. Part D Transp. Environ..

[83] Torres S., Lalanne F., del Canto G., Morales F., Bustos-Jimenez J., Reyes P. (2015). BeCity: Sensing and sensibility on urban cycling for smarter cities. Proceedings of the 34th International Conference of the Chilean Computer Science Society (SCCC).

[84] Manum B., Nordström T., Gil J., Nilsson L., Marcus L. Modelling bikeability; Space syntax based measures applied in examining speeds and flows of bicycling in Gothenburg. Proceedings of the 11th International Space Syntax Symposium;.

[85] Crockett J., O’Hare S., Barritt A., Gordon A., Inayathusein A., Forrest R. (2016). In the Peleton or in the Break—Factors Affecting Cyclists Route Choice in London. European Transport Conference.

[86] Sun Y., Mobasheri A. (2017). Utilizing Crowdsourced Data for Studies of Cycling and Air Pollution Exposure: A Case Study Using Strava Data. Int. J. Environ. Res. Public Health.

[87] Norman G., Kesha N. (2015). Using smartphones for cycle planning. Institution of Professional Engineers New Zealand (IPENZ) Transportation Conference.

[88] Musakwa W., Selala K.M. (2016). Mapping cycling patterns and trends using Strava Metro data in the city of Johannesburg, South Africa. Data Br..

[89] Bergman C., Oksanen J. (2016). Conflation of OpenStreetMap and Mobile Sports Tracking Data for Automatic Bicycle Routing. Trans. GIS.

[90] Schweizer J., Bernardi S., Rupi F. (2016). Map-matching algorithm applied to bicycle global positioning system traces in Bologna. IET Intell. Transp. Syst..

[91] Baker K., Ooms K., Verstockt S., Brackman P., De Maeyer P., Van de Walle R. (2017). Crowdsourcing a cyclist perspective on suggested recreational paths in real-world networks. Cartogr. Geogr. Inf. Sci..

[92] Leao S.Z., Pettit C., Namazi-Rad M.-R., Padgham L., Perez P., Nagel K., Bazzan A. (2017). Agent Based Modelling of Urban Systems. Lecture Notes in Computer Science.

[93] Ton D., Cats O., Duives D., Hoogendoorn S. (2017). How Do People Cycle in Amsterdam? Estimating cyclists’ route choice determinants using GPS data from an urban area. Transp. Res. Rec. J. Transp. Res. Board.

[94] Bussche D., Van de Coevering P. (2015). BikePRINT—In Depth Analysis of Cyclist Behaviour and Cycle Network Performance Using Extensive Gps-Track Data. European Transport Conference.

[95] Lee D., Park S., Hahn M. (2014). A study on bicycle crash risk based on GPS trajectories collected from bidirectional traffic. Proceedings of the 2014 IEEE Asia Pacific Conference on Circuits and Systems (APCCAS).

[96] Assemi B., Schlagwein D., Safi H., Mesbah M., Wirtz G., Townend P. (2015). Crowdsourcing as a Method for the Collection of Revealed Preferences Data. 2015 IEEE Symposium on Service-Oriented System Engineering.

[97] Lott D.F., Tardiff T., Lott D.Y. (1978). Evaluation by experienced riders of a new bicycle lane in an Established Bikeway System. Transp. Res. Rec. J. Transp. Res. Board.

[98] Van Schagen I.N.L.G. (1990). Pedestrians and Pedal Cyclists in a British, Dutch and Swedish Modelling Area.

[99] Kang L., Fricker J.D. (2013). Bicyclist commuters’ choice of on-street versus off-street route segments. Transportation.

[100] Van Maarseveen M., Jansen G.R.M., Bovy P.H.L. (1985). Estimating OD Tables Using Empirical Route-Choice Information with Application to Bicycle Traffic. Transp. Res. Rec. J. Transp. Res. Board.

[101] Wilmink A., Hartman J.B. (1987). Evaluation of the Delft Bicycle Network Plan. Final Summary Report.

[102] Aultman-Hall L., Hall F.L., Baetz B. (1997). Analysis of bicycle commuter routes using geographic information systems: implications for bicycle planning. Transp. Res. Rec. J. Transp. Res. Board.

[103] Aultman-Hall L. (1996). Commuter Bicycle Route Choice: Analysis of Major Determinants and Safety Implications,. Ph.D. Thesis.

[104] Howard C., Burns E. (2001). Cycling to Work in Phoenix: Route Choice, Travel Behavior, and Commuter Characteristics. Transp. Res. Rec. J. Transp. Res. Board.

[105] Boettge B., Hall D., Crawford T. (2017). Assessing the Bicycle Network in St. Louis: A PlaceBased User-Centered Approach. Sustainability.

[106] Manton R., Rau H., Fahy F., Sheahan J., Clifford E. (2016). Using mental mapping to unpack perceived cycling risk. Accid. Anal. Prev..

[107] Manum B., Nordström T., Kim Y.O., Park H.T. (2013). Integrating Bicycle Network Analysis in Urban Design: Improving bikeability in Trondheim by combining space syntax and GIS-methods using the place syntax tool. International Space Syntax Symposium.

[108] Hyodo T., Suzuki N., Takahashi K. (2000). Modeling of Bicycle Route and Destination Choice Behavior for Bicycle Road Network Plan. Transp. Res. Rec. J. Transp. Res. Board.

[109] Suzuki K., Kanda Y., Doi K., Tsuchizaki N. (2012). Proposal and Application of a New Method for Bicycle Network Planning. Procedia Soc. Behav. Sci..

[110] Yang C., Mesbah M., Bunker J.M., Burke M. (2013). Route Choice Behaviour of Cyclists by Stated Preference and Revealed Preference. Australasian Transport Research Forum.

[111] Gamble J., Snizek B., Nielsen T.S. (2017). From people to cycling indicators: Documenting and understanding the urban context of cyclists’ experiences in Quito, Ecuador. J. Transp. Geogr..

[112] Wang J.Y.T., Mirza L., Cheung A.K.L., Moradi S., Hughes B., Petkoff I. (2012). Transforming Auckland into a bicycle-friendly city: Understanding factors influencing choices of cyclists and potential cyclists. Australasian Transport Research Forum.

[113] Snizek B., Sick Nielsen T.A., Skov-Petersen H. (2013). Mapping bicyclists’ experiences in Copenhagen. J. Transp. Geogr..

[114] Kobas G.V., Drury C.G. (1976). The Bicyclist’s Exposure to Risk. Hum. Factors Ergon. Soc. Annu. Meet..

[115] Krizek K.J., El-Geneidy A., Thompson K. (2007). A detailed analysis of how an urban trail system affects cyclists’ travel. Transportation.

[116] Larsen J., El-Geneidy A. (2011). A travel behavior analysis of urban cycling facilities in Montréal, Canada. Transp. Res. Part D Transp. Environ..

[117] Standen C., Crane M., Collins A., Greaves S., Rissel C. (2017). Determinants of mode and route change following the opening of a new cycleway in Sydney, Australia. J. Transp. Health.

[118] Winters M., Teschke K., Grant M., Setton E., Brauer M. (2010). How Far Out of the Way Will We Travel?. Transp. Res. Rec. J. Transp. Res. Board.

[119] González F., Melo-Riquelme C., de Grange L. (2016). A combined destination and route choice model for a bicycle sharing system. Transportation.

[120] Salmon R., Chinnock C., Duckenfield T. (2012). Cycle Route Choice. Final Survey and Model Report.

[121] Van Duppen J., Spierings B. (2013). Retracing trajectories: The embodied experience of cycling, urban sensescapes and the commute between “neighbourhood” and “city” in Utrecht, NL. J. Transp. Geogr..

[122] Barratt P. (2017). Healthy competition: A qualitative study investigating persuasive technologies and the gamification of cycling. Health Place.

[123] Zacharias J., Zhang R. (2016). Revealed Bicyclist Route Preferences and Street Conditions. Transp. Res. Rec. J. Transp. Res. Board.

[124] Simpson P. (2017). A sense of the cycling environment: Felt experiences of infrastructure and atmospheres. Environ. Plan. A.

[125] Johnson M., Charlton J., Oxley J., Newstead S. (2010). Naturalistic cycling study: Identifying risk factors for on-road commuter cyclists. Ann. Adv. Automot. Med..

[126] Stewart K., McHale A. (2014). Cycle lanes: their effect on driver passing distances in urban areas. Transport.

[127] De Leeuw G., de Kruijf J. (2015). The Act of (Future) Cycling: Testing Urban Designs and Conducting Research in Virtual Reality. European Transport Conference.

[128] Hirose S., Kitamura Y. (2015). Preliminary evaluation of virtual cycling system using google street view. Persuas. Technol..

[129] Heinen E., van Wee B., Maat K. (2010). Commuting by Bicycle: An Overview of the Literature. Transp. Rev..

